# A *Csf1r*-EGFP Transgene Provides a Novel Marker for Monocyte Subsets in Sheep

**DOI:** 10.4049/jimmunol.1502336

**Published:** 2016-08-12

**Authors:** Clare Pridans, Gemma M. Davis, Kristin A. Sauter, Zofia M. Lisowski, Yolanda Corripio-Miyar, Anna Raper, Lucas Lefevre, Rachel Young, Mary E. McCulloch, Simon Lillico, Elspeth Milne, Bruce Whitelaw, David A. Hume

**Affiliations:** * The Roslin Institute, University of Edinburgh, Midlothian EH25 9RG, United Kingdom; and; † Royal (Dick) School of Veterinary Studies, University of Edinburgh, Midlothian EH25 9RG, United Kingdom

## Abstract

Expression of Csf1r in adults is restricted to cells of the macrophage lineage. Transgenic reporters based upon the Csf1r locus require inclusion of the highly conserved Fms-intronic regulatory element for expression. We have created *Csf1r*-EGFP transgenic sheep via lentiviral transgenesis of a construct containing elements of the mouse Fms-intronic regulatory element and *Csf1r* promoter. Committed bone marrow macrophage precursors and blood monocytes express EGFP in these animals. Sheep monocytes were divided into three populations, similar to classical, intermediate, and nonclassical monocytes in humans, based upon CD14 and CD16 expression. All expressed EGFP, with increased levels in the nonclassical subset. Because *Csf1r* expression coincides with the earliest commitment to the macrophage lineage, *Csf1r*-EGFP bone marrow provides a tool for studying the earliest events in myelopoiesis using the sheep as a model.

## Introduction

The development of macrophages requires signaling from the CSF1 receptor (CSF1R) initiated by one of two ligands, CSF1 and IL-34 (1, 2). Expression of *Csf1r* mRNA in adult mice is restricted to cells of the macrophage lineage (3). The lineage-restricted expression of *Csf1r* provides the basis for the development of macrophage-specific transgenes. MacGreen (*Csf1r*-EGFP) mice were created previously by placing EGFP expression under the control of a 7.2-kb region of the murine *Csf1r* proximal promoter. Expression of the transgene is dependent on the inclusion of the highly conserved Fms-intronic regulatory element (FIRE), located in the second intron of *Csf1r* (4). MacGreen mice express EGFP in the same locations as the endogenous gene and have been widely used to visualize macrophages (5–8). Embryonic expression of the MacGreen transgene is restricted to macrophages and trophoblasts (4), and expression of the latter relies on a conserved 150-bp region of the *Csf1r* promoter. The trophoblast promoter region was omitted from the construct used in the generation of the MacBlue (*Csf1r*-Gal4VP16/UAS-ECFP) transgenic mice (9). In these mice, ECFP is absent from osteoclasts and the majority of tissue macrophages, but expression is retained in microglia and Langerhans cells (10). MacGreen and MacBlue mice were created by standard pronuclear injection techniques, a relatively inefficient gene delivery tool when compared with lentiviral-based methods (11). To increase the efficiency of macrophage-specific transgene delivery, we created a lentiviral vector containing elements of murine *Csf1r* (12). The *Csf1r* promoter (0.5-kb including the start codon) was fused to the coding sequence of *EGFP* and cloned upstream of the intronic region containing FIRE. The lentivirus (*Csf1r*-EGFP-FIRE) was capable of directing EGFP expression in mouse, rat, human, pig, cow, sheep, and even chicken macrophages in vitro. This remarkable cross-species reactivity led us to produce *Csf1r*-EGFP sheep via injection of lentivirus into the perivitelline space of fertilized embryos. This is a highly efficient method to generate transgenic sheep (13). In addition to being economically important, sheep are increasingly being used as models for human diseases such as Huntington disease (14) and Batten disease (15). They themselves are susceptible to a variety of diseases such as paratuberculosis or Johne disease where macrophages are known to play a role (16). Although ovine macrophages have been studied in response to infection (17, 18), little is known about the characteristics of their monocytic precursors. In contrast, monocyte subpopulations have been well described in human, mouse, and pig (reviewed in Ref. 19). In this article, we describe the generation and characterization of *Csf1r*-EGFP sheep. Monocytes and their precursors express EGFP, yet expression of the transgene is downregulated in differentiated macrophages. These sheep provide a useful tool for studies involving subpopulations of monocytes and their precursors.

## Materials and Methods

### Animals

Approval was obtained from The Roslin Institute’s and the University of Edinburgh’s Protocols and Ethics Committees. The experiments were carried out under the authority of a U.K. Home Office Project License under the regulations of the Animals (Scientific Procedures) Act 1986. Sheep and mice were euthanized via captive bolt or CO_2_ asphyxiation, respectively.

### Constructs and lentivirus preparation

Constructs and preparation of lentivirus (*Csf1r*:EGFP-FIRE) was performed as described by Pridans et al. (12). The viral titer was 2.2 × 10^7^ transducing U/ml as assayed by endpoint dilution on the D-17 cell line.

### Embryo manipulation and generation of transgenic sheep

Zygotes were obtained via methods described by Ritchie et al. (20) using abattoir-derived oocytes and frozen sperm from a Shetland ram. The lentivirus was injected into the perivitelline space of zygotes that were developed in vitro until the blastocyst stage (days 6–7), at which point they were transplanted into the uterine horn of recipient ewes. The integration of the transgene in lambs was investigated by PCR analysis of ear biopsy DNA, amplifying *EGFP* using primers 5′-GCACGACTTCTTCAAGTCCGCCATGCC-3′ (forward) and 5′-GCGGATCTTGAAGTTCACCTTGATGCC-3′ (reverse). Plasmid DNA (*Csf1r*:EGFP-FIRE) and genomic DNA from a wild type sheep were used as positive and negative controls, respectively.

### Isolation of PBMCs from sheep

Bags containing citrate phosphate dextrose adenine (Infusion Concepts, Sowerby Bridge, U.K.) were used to collect blood from live animals <1 y of age. Blood was layered onto an equal volume of Lymphoprep (Axis-Shield, Oslo, Norway) and centrifuged at 1200 × *g* for 25 min with no brake. The PBMC layer was washed in an equal volume of PBS containing 2% FCS (FACS buffer). Any contaminating RBCs were removed by resuspending the pellet in 1 ml RBC lysis buffer (BioLegend, London, U.K.) and immediately topping up to 50 ml with FACS buffer. After centrifugation (400 × *g*, 5 min) cells were washed once with buffer.

### Bone marrow and alveolar macrophage isolation from sheep

Bone marrow (BM) and alveolar macrophages (AMs) were isolated as described by Kapetanovic et al. (21), except RBC lysis buffer (BioLegend) was used and the medium was RPMI 1640 supplemented with 20% sheep serum, 100 U/ml penicillin, 100 μg/ml streptomycin, and 1 mmol/l GlutaMAX (Life Technologies, Paisley, U.K.).

### In vitro differentiation of sheep macrophages

Freshly isolated BM and PBMCs were cultured on bacteriological plates at a density of 2 × 10^5^ and 3 × 10^5^ cells/cm^2^, respectively. Complete medium was supplemented with 10^4^ U/ml (100 ng/ml) recombinant human CSF1 (rhCSF1; a gift from Chiron, Emeryville, CA). Fresh media containing rhCSF1 was added on day 4 and cells analyzed on day 7.

### Phagocytosis assays using macrophages and whole blood from sheep

Freshly isolated PBMCs and BM from *Csf1r*-EGFP sheep were cultured in two-well Lab-Tek chamber slides (Nunc) at a density of 5 × 10^5^ cells/cm^2^ as described earlier. Zymosan A *Saccharomyces cerevisiae* BioParticles labeled with Alexa Fluor (AF) 594 (Thermo Fisher Scientific, Waltham, MA) were added at 8 × 10^5^ particles/well and incubated for 2 h at 37°C. After addition of ice-cold PBS, cells were washed four times with PBS and fixed in 4% paraformaldehyde for 20 min at room temperature. Cells were washed in PBS and viewed by confocal microscopy. For comparison of phagocytic activity between wild type and *Csf1r*-EGFP sheep, whole blood was collected in EDTA tubes and assays performed based on a modified protocol from Bicker et al. (22). pHrodo Red *Escherichia coli* BioParticles (Thermo Fisher) were added to an equal volume of whole blood and centrifuged at 34 × *g*, 37°C for 1 h. A control blood sample containing the BioParticles was left on ice during this time. Samples were incubated on ice for 10 min and then stained with mouse anti-human CD14 AF647 (clone TÜK4, 1:20; AbD Serotec, Kidlington, U.K.) for 1 h on ice. Blood was then prepared for flow cytometry using Dako Uti-lyse erythrocyte lysing solution according to instructions (Dako, Denmark).

### Microscopy

Cells were imaged using a LSM710 confocal microscope and ZEN software (Zeiss, Cambridge, U.K.) or via standard light microscopy.

### Flow cytometry

Whole blood or PBMCs (freshly isolated or cryopreserved) from sheep were used for analysis by flow cytometry on a FACSCalibur or Fortessa X20 (BD, Oxford, U.K.). PBMCs were washed and stained in PBS containing 2% FCS at 4°C. Propidium iodide (1 μg/ml; Sigma, Dorset, U.K.) or SYTOX Blue (Life Technologies) was used to exclude dead cells. Cells were stained with the following primary Abs: mouse anti-human CD16 (clone KD1, 1:100; LifeSpan BioSciences, Seattle, WA), mouse anti-bovine CD14 (clone CC-G33, 1:400), anti-human CD14 (clone TÜK4, 1:20), mouse anti-bovine CD172a (clone CC149, 1:200; all from AbD Serotec), and rat anti-sheep MHC class II (clone SW73.2 [23] ascites 1:4000). Isotype controls were used at the same concentrations as primary Abs: mouse IgG2a, IgG1-RPE, IgG2b (AbD Serotec) and rat IgG2a (BioLegend). Secondary Abs used were anti-mouse IgG2a-allophycocyanin (1:200), IgG1-RPE (1:800), IgG1-allophycocyanin (1:200), IgG2b-RPE (1:400) and anti-rat IgG2a AF647 (1:4000; all from BioLegend). Blood was collected in EDTA tubes from live sheep or from mice via cardiac bleeds. Blood was prepared for flow cytometry using Dako Uti-lyse erythrocyte lysing solution (Dako) using Zombie Violet (BioLegend) to exclude dead cells. CSF1-Fc (6) was labeled with an AF647 labeling kit (Thermo Fisher Scientific). Analysis was performed with FlowJo software (FlowJo, Ashland, OR).

### Cell sorting and cytospins

Freshly isolated BM and PBMCs were used for sorting EGFP^+^ populations on a FACSAria III cell sorter (BD). SYTOX Blue (Life Technologies) was used to exclude dead cells. Cells were spotted on polylysine slides with a cytocentrifuge (Shandon, Runcorn, U.K.), stained with Leishman’s stain and mounted with DPX mounting media.

### cDNA synthesis and PCR

Total RNA from sheep BM-derived macrophages (BMDMs) was isolated using TRIzol (Invitrogen), and the aqueous phase was purified with an RNeasy Mini kit (Qiagen). cDNA was prepared as in Pridans et al. (12). For standard PCR, *EGFP* and ovine *Csf1r* were amplified with Invitrogen Taq polymerase using the following oligonucleotides: *EGFP*, 5′-CCACAAGTTCAGCGTGTCC-3′ and 5′-CTTGTACAGCTCGTCCATGC-3′; *Csf1r*, 5′-AGTCCTGACCCTCAAACTCG-3′ and 5′-GGGTGAGCTTGGAGGTGTAT-3′. For quantitative PCR, cDNA was amplified with Power SYBR Green PCR Master Mix using the 7500 fast Real Time PCR system (Applied Biosystems, Thermo Fisher Scientific). The oligonucleotides used were: *Hprt* 5′-GACACTGGGAAGACAATGCA-3′ and 5′-GTCCTTTTCACCAGCAAGCT-3′; *Csf1r* 5′-TGGTGAAGTCCCTCAGCATC-3′ and 5′-CCTTGAATCCGCACCAGTTC-3′. Primer efficiency was validated with a standard curve of four serial dilution points (efficiency ranging between −3.28 and −3.38), and tests for nonamplification of genomic DNA were carried out systematically. Data were normalized according to the ΔCq model (24).

### Western blot

Radioimmunoprecipitation assay lysates (50 mM of Tris pH 7.0, 150 mM of NaCl, 0.1% SDS, 1% IGEPAL CA-630, 1.27 mM of sodium deoxycholate) were prepared from CSF1-starved BMDMs (1 ml per 5 × 10^7^ cells) and EGFP^+^ RAW264.7 cells (12). Samples were mixed with 4× loading dye (0.25 M Tris-HCl pH 6.8, 8% SDS, 30% glycerol, 0.02% bromophenol blue, 10% 2-ME), heated for 5 min at 95°C, run on a 4–12% gradient SDS-PAGE gel, and transferred onto a polyvinylidene difluoride membrane as per Bio-Rad instructions. The membrane was blotted using a GFP Tag mAb (Life Technologies) and β-actin Ab (C4; Santa Cruz Biotechnology). Secondary Abs were anti-rabbit and -mouse IgG HRP (Cell Signaling Technology).

## Results

### Production and screening of *Csf1r*-EGFP transgenic sheep

**FIGURE 1.**
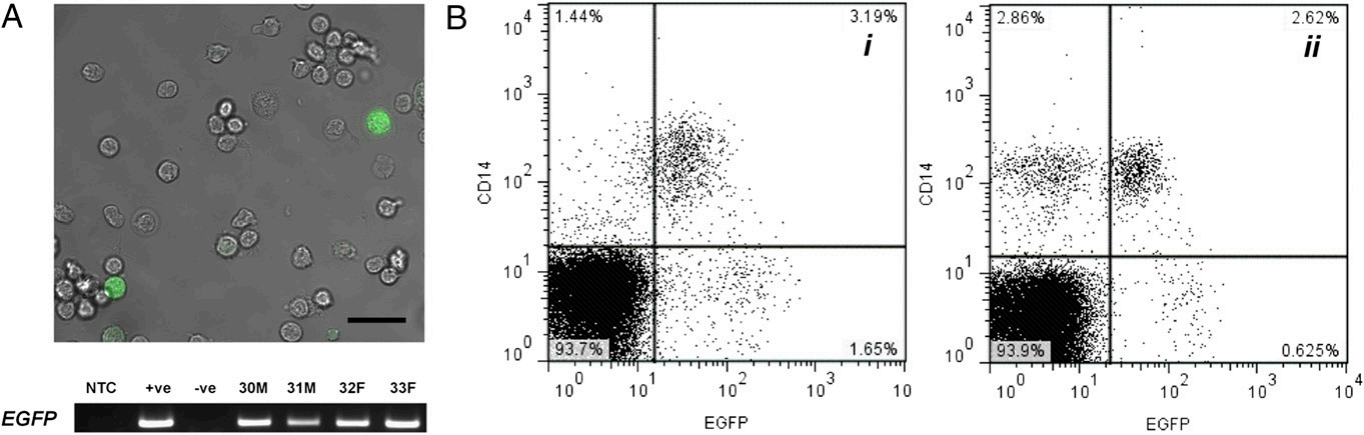
Monocytes of *Csf1r*-EGFP founder sheep express EGFP. (**A**) Confocal microscopy of *Csf1r*-EGFP sheep PBMCs (scale bar, 20 μm, representative of four sheep) and *EGFP* PCR using genomic DNA from founder animals. (**B**) Representative flow cytometry analysis of CD14 and EGFP expression in PBMCs from two founder sheep (**i** and **ii**). Cells were gated on lymphocytes and monocytes based on FSC/SSC. Propidium iodide was used to exclude dead cells. NTC, no template control; +ve, plasmid DNA; −ve, negative control sheep genomic DNA.

Our recent in vitro study of a lentiviral vector containing control elements of murine *Csf1r* revealed macrophage-specific gene reporter expression in multiple species including sheep (12). Zygotes were injected with lentivirus (*Csf1r*:EGFP-FIRE [12]) and developed to blastocysts in vitro to create germline transgenic *Csf1r*-EGFP sheep. Eight recipient ewes were transplanted with either two or three blastocysts, which resulted in three pregnancies. Four founder lambs (two male and two female) were born and all were positive for the transgene (EGFP) via confocal microscopy and PCR (Fig. 1A). The LPS receptor CD14 is a monocyte marker (25) and was used to initially screen the founders by flow cytometry. Two reporter gene expression patterns were observed: all CD14^+^ monocytes expressed EGFP in two of the lambs (Fig. 1Bi), whereas the other two lambs had a population of CD14^+^EGFP^−^ monocytes (Fig. 1Bii). There was also a small percentage of EGFP^hi^ cells that did not express CD14, which is discussed later (Fig. 1B). The two founder males (as shown in Fig. 1B) were bred to wild type Blackface ewes. Seven lambs were born to the CD14^+^EGFP^+^ founder (Fig. 1Bi), and six were positive for the transgene via PCR. Five of these expressed detectable EGFP in blood in a preliminary screen by flow cytometry. There was a lower percentage of offspring with EGFP^+^ monocytes born to the CD14^+/−^EGFP^+^ founder (Fig. 1Bii). Of the 13 lambs born, 10 were *EGFP*^+^ via PCR, yet only three expressed EGFP via flow cytometry. Although this founder ram had a population of CD14^+^EGFP^−^ monocytes (Fig. 1Bii), analysis of the offspring’s blood revealed all CD14^+^ monocytes expressed EGFP (data not shown). At the time of this article’s publication, lambs were born from the F1 generation (*Csf1r*-EGFP male bred with wild type ewe) and had the same phenotype as the founders in Fig. 1Bi. All EGFP^+^ offspring were used in subsequent experiments.

### Analysis of BM from *Csf1r*-EGFP sheep

**FIGURE 2.**
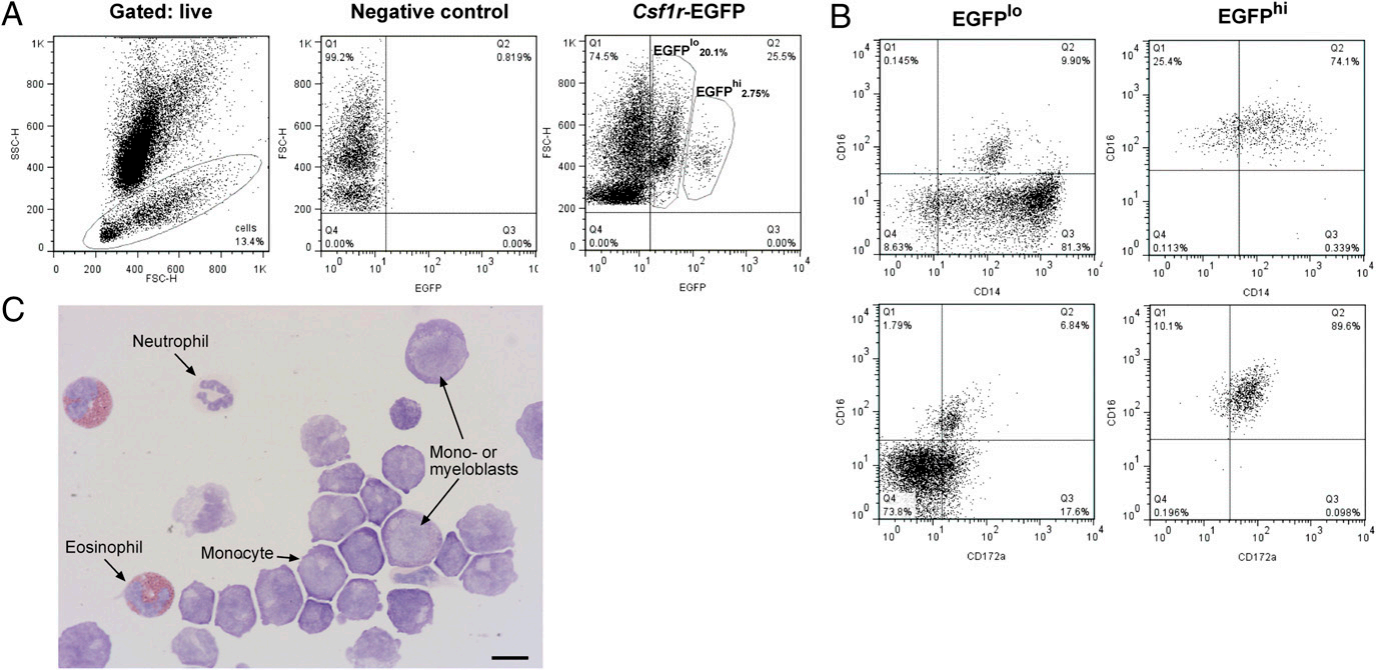
BM of *Csf1r*-EGFP sheep contains a heterogeneous EGFP^+^ population of cells. BM from *Csf1r*-EGFP sheep was isolated from ribs and analyzed via flow cytometry. (**A**) Gating strategy. Dead cells were excluded with propidium iodide staining and granulocytes excluded via FSC/SSC. EGFP^lo^ and EGFP^hi^ were selected once quadrants were set with negative control BM. (**B**) Analysis of CD14, CD16, and CD172a expression in GFP^lo^ and GFP^hi^ cells. Quadrants were set using isotype controls for each population. (**C**) Leishman-stained EGFP^+^ cells (combined EGFP^lo^ and EGFP^hi^). Scale bar, 10 μm. Dot plots and Leishman staining of EGFP^+^ cells representative of five and two sheep, respectively. Leishman staining was representative of 15 images per animal.

BM contains hemopoietic stem cells that create monocytes via a series of progenitor cells (26). To determine whether EGFP was expressed in the progenitors of monocytes within the BM of *Csf1r*-EGFP sheep, we isolated cells from ribs and analyzed them by flow cytometry. To exclude autofluorescent eosinophils (27) and other granulocytes, we gated the marrow population on lymphocytes/monocytes based on forward light scatter (FSC) and side scatter of light (SSC) profiles (Fig. 2A). The small mononuclear population had an average of 28.5% EGFP^+^ cells (*n* = 5) and could be divided into EGFP^hi^ and EGFP^lo^ populations. There was an average of 1.7% EGFP^hi^ cells that were CD16^+^CD172a^+^ and expressed an increasing level of CD14. The EGFP^lo^ cells were more heterogeneous. There was a wider range of CD14 expression than the EGFP^hi^ cells, and only a small percentage (<10%) also expressed CD16 and CD172a (Fig. 2B). Leishman staining of sorted EGFP^+^ cells revealed that the majority of cells were monocytoid. The large blast cells were characteristic of myeloblasts and monoblasts (28) (Fig. 2C).

### Analysis of PBMCs from *Csf1r*-EGFP sheep

**FIGURE 3.**
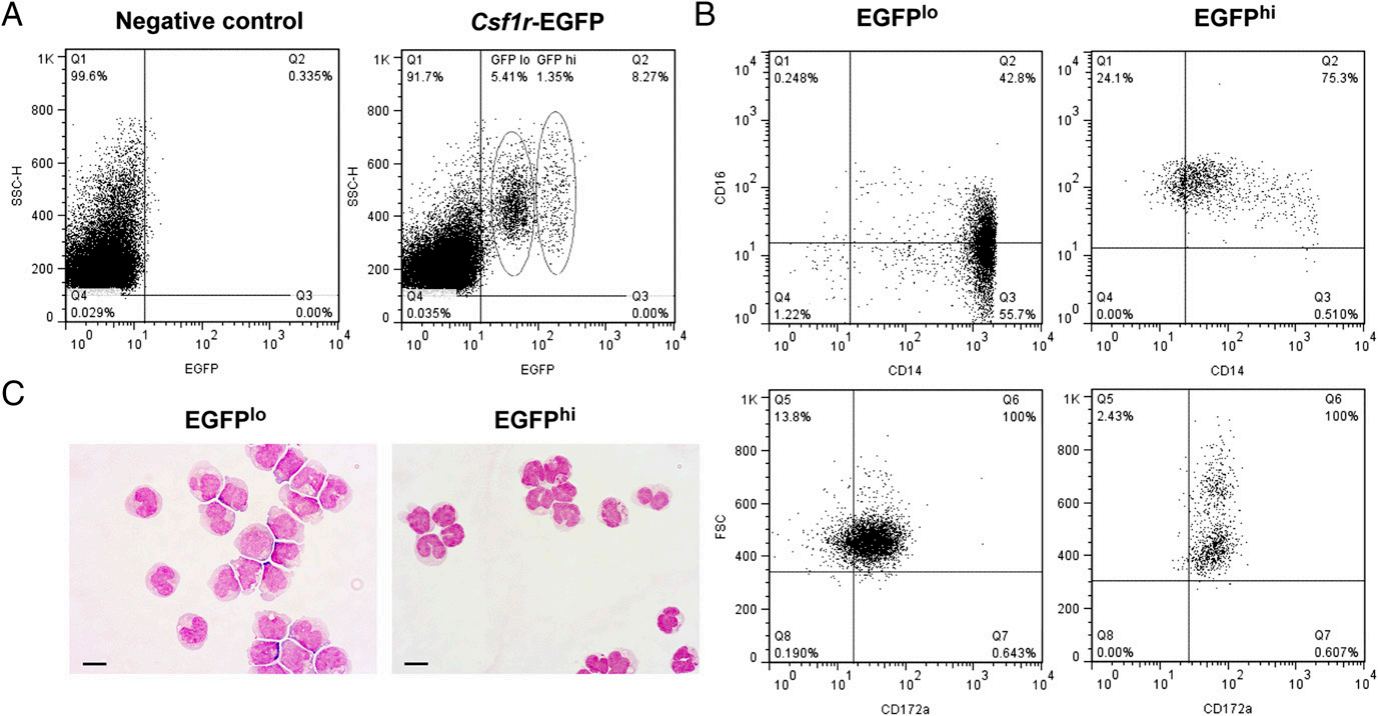
Monocytes are EGFP^+^ in *Csf1r*-EGFP sheep. Peripheral blood monocytes were isolated from the blood of live animals and analyzed via flow cytometry. (**A**) EGFP^+^ gating strategy. (**B**) Expression of CD14, CD16, and CD172a in EGFP^lo^ and EGFP^hi^ PBMCs. Quadrants were set using isotype controls for each population. (**C**) Leishman-stained EGFP^+^ cells. Scale bars, 10 μm. Dot plots and Leishman staining of EGFP^+^ cells are representative of nine and three sheep, respectively. Leishman staining was representative of 10 images per animal.

Peripheral blood monocytes in mice, humans, and pigs can be divided into functional subsets based upon expression of surface markers including CD14, CD16, CX3CR1, and CD163 (29–31). Differentiation of these subsets appears to be controlled by the macrophage growth factor, CSF1 (1, 2). Monocytes in MacGreen mice are EGFP^+^ (4). To analyze EGFP expression in monocytes from the sheep, blood samples were taken from live animals and PBMCs purified using a density gradient. Sheep are known to have a lower percentage of blood monocytes compared with humans or mice (32), and an average of 4.2% of PBMCs expressed EGFP (range 1.2–9.9%). EGFP^+^ PBMCs could be divided into EGFP^hi^ and EGFP^lo^ populations (Fig. 3A). The EGFP^lo^ cells were CD14^++^CD16^+/−^, characteristic of human classical and intermediate monocytes (33) whereas the EGFP^hi^ cells displayed the nonclassical monocyte phenotype (CD14^+^ CD16^++^). Both populations expressed CD172a (Fig. 3B), and FSC could be used to separate the EGFP^hi^ cells into two distinct populations. Nonclassical monocytes are known to be smaller than classical monocytes (34), and Leishman staining on sorted cells revealed that both subsets presented monocyte morphology (Fig. 3C). CD16^+^ human monocytes express higher levels of *Csf1r* mRNA than CD16^−^ monocytes (35), and this could be reflected in the increase in EGFP expression in CD16^+^ monocytes from *Csf1r*-EGFP sheep.

### Downregulation of the EGFP transgene in macrophages of sheep

**FIGURE 4.**
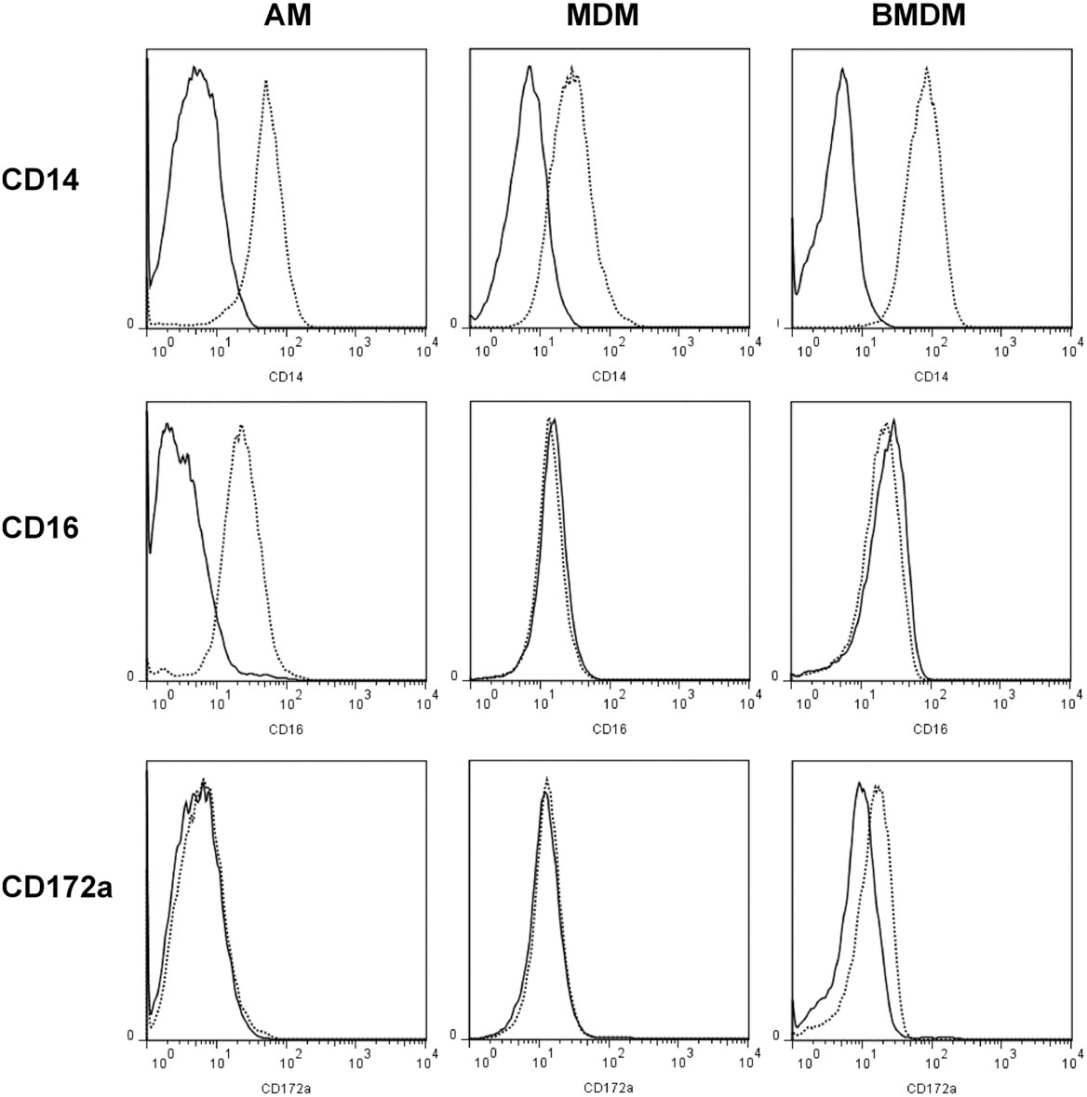
Flow cytometry analysis of AMs, MDMs, and BMDMs from sheep. A lung lavage was performed to isolate AMs. Macrophages were differentiated from PBMCs and BM for 7 d with rhCSF1. Dead cells were excluded with propidium iodide. Solid lines represent isotype controls. Histograms are representative of three (AM and MDM) or five (BMDM) sheep.

Treatment of BM cells or blood monocytes from other species with CSF1 can promote maturation into macrophages in vitro (4, 21, 36). To analyze EGFP expression in macrophages from *Csf1r*-EGFP sheep, we isolated AMs via lung lavage and PBMCs/BM were differentiated in vitro in the presence of rhCSF1. The resulting cell populations were analyzed for surface expression of CD14, CD16, and CD172a. All three cell populations retained high levels of surface CD14, but they differed in expression of CD16 and CD172a. AMs were the only cell type to express CD16, and BMDMs expressed very low levels of CD172a (Fig. 4). In all of these populations of cells, detectable expression of the EGFP transgene was extinguished. Hence the transgene provides a novel marker for blood monocytes that is lost from mature macrophages.

**FIGURE 5.**
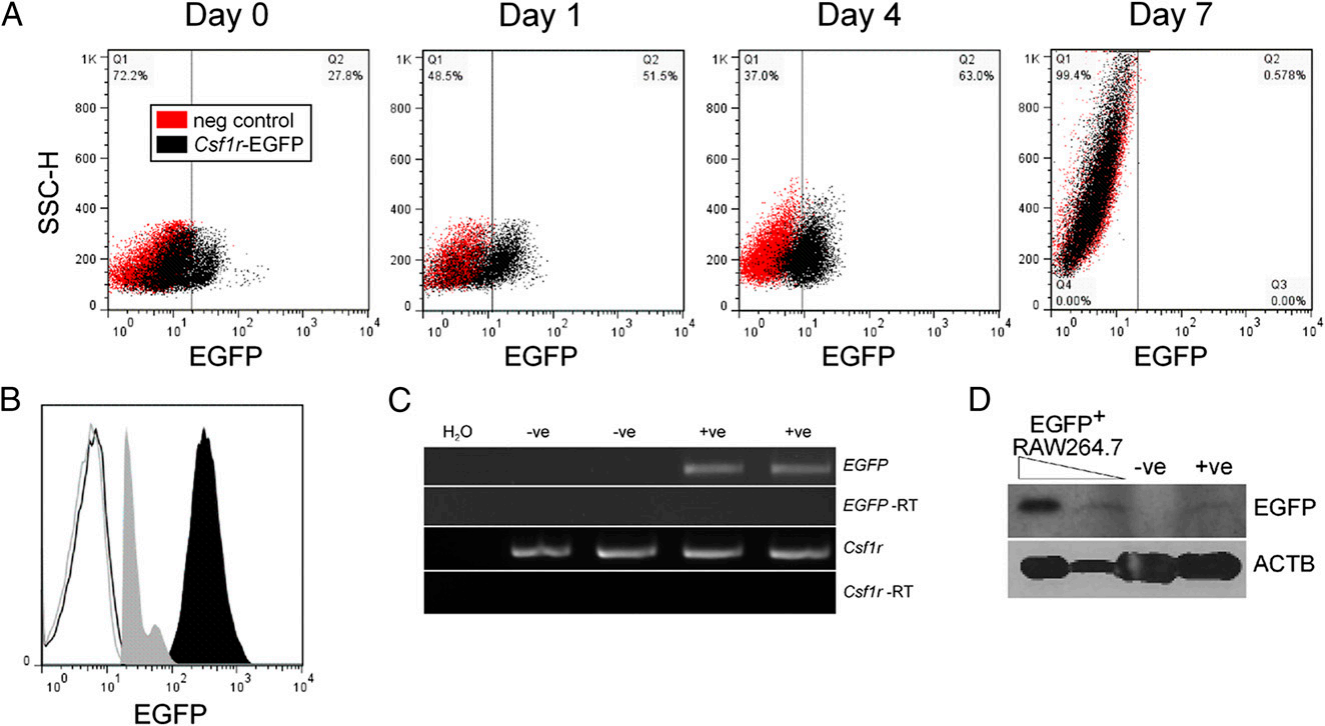
Downregulation of EGFP expression during macrophage differentiation. (**A**) Cryopreserved BM from wild type and *Csf1r*-EGFP sheep were cultured in rhCSF1 and then analyzed via flow cytometry for EGFP. Monocyte precursors were gated via FSC/SSC profiles and dead cells excluded with propidium iodide. Dot plots are representative of two sheep. (**B**) Flow cytometry analysis of EGFP expression in monocytes from *Csf1r*-EGFP mice (solid black) and sheep (solid gray) compared with wild type animals. Monocytes were gated via FSC/SSC profiles. Histograms are representative of three animals. (**C**) mRNA was prepared from wild type (−ve) and *Csf1r*-EGFP (+ve) BMDMs and used in RT-PCR to analyze *EGFP* expression. Each image is representative of three experiments. (**D**) Protein lysates from BMDMs were analyzed via Western blot for EGFP and ACTB expression. Blot is representative of two experiments.

To determine when EGFP expression was lost during differentiation, we cultured BM cells in rhCSF1 and analyzed EGFP expression via flow cytometry (Fig. 5A). After 24 h in culture with rhCSF1, the EGFP^hi^ population was no longer visible and 50% of the cells expressed low levels of EGFP. This pattern of expression continued until day 4, when there was a slight increase in the granularity of the cells. After 7 d in culture, once the BM had differentiated into macrophages, EGFP expression was no longer detected. Expression of *Csf1r* mRNA is downregulated by CSF1 (37), and macrophages express lower levels of *Csf1r* compared with monocytes (38). Because the levels of EGFP in *Csf1r*-EGFP sheep are very low, downregulation of *Csf1r* during differentiation would likely result in loss of detectable EGFP. Indeed, comparison of EGFP in PBMCs from *Csf1r*-EGFP sheep and mice demonstrates the sheep express 1 log-fold less EGFP (Fig. 5B) and still express *EGFP* mRNA in BMDMs (Fig. 5C). Mature macrophages in *Csf1r*-EGFP mice are easily detected via FACS and microscopy (4). Notwithstanding the lower expression, EGFP could still be detected in the sheep macrophages via Western blot (Fig. 5D). As we have previously shown that *Csf1r*-EGFP lentivirus is capable of transducing primary sheep macrophages in vitro to generate an EGFP^+^ population (12), producing *Csf1r*-EGFP sheep with a higher titer of lentivirus may result in higher levels of EGFP^+^ in macrophages.

### Functional analyses of monocytes and macrophages from *Csf1r*-EGFP sheep

**FIGURE 6.**
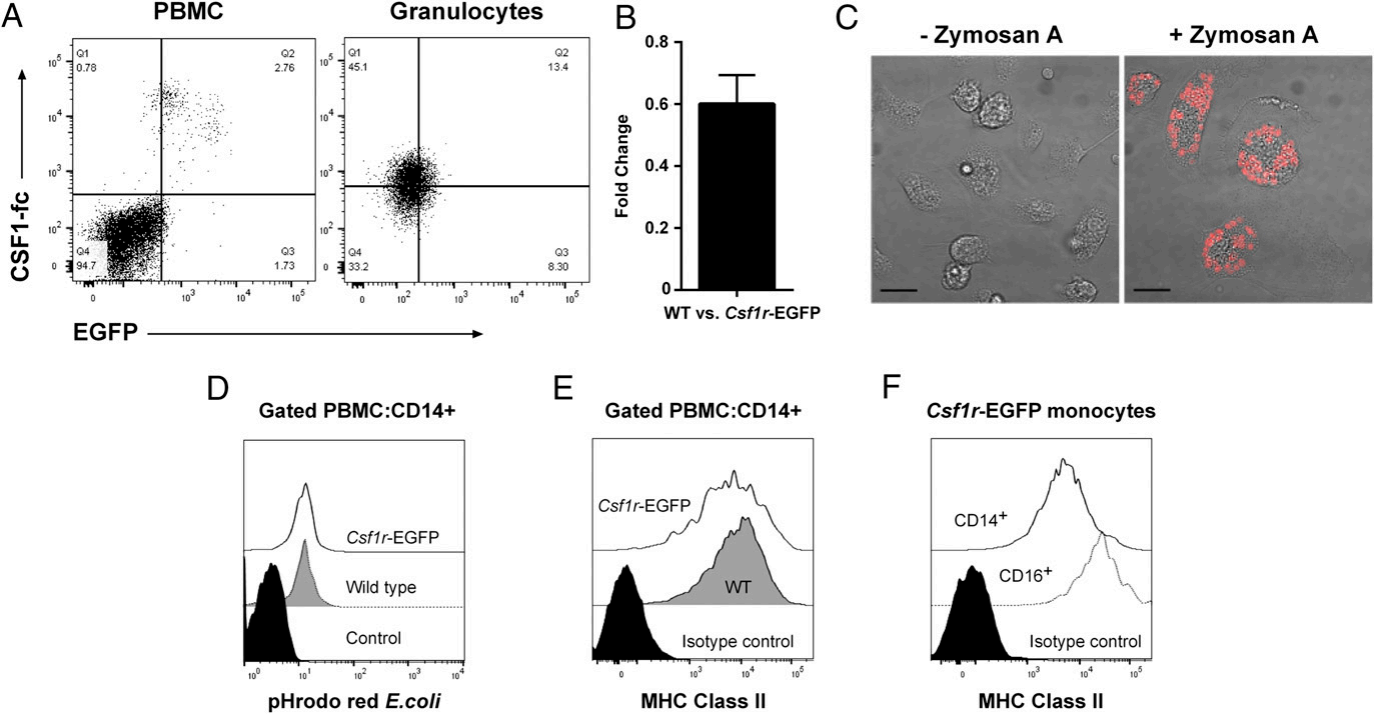
(**A**) Freshly isolated blood from wild type and *Csf1r*-EGFP sheep was analyzed via flow cytometry for binding of CSF1-Fc and EGFP expression. PBMCs and granulocytes were gated via FSC/SSC profiles and dead cells excluded with Zombie Violet. Quadrants were set with wild type sheep blood. Dot plots are representative of three *Csf1r*-EGFP sheep and two repeat experiments. (**B**) Quantitative PCR was used to determine the fold change of *Csf1r* expression between wild type and *Csf1r*-EGFP sheep BMDMs (*n* = 4 per group). (**C**) PBMCs from *Csf1r*-EGFP sheep were differentiated into macrophages with rhCSF1. Phagocytosis assays performed with Zymosan A *S. cerevisiae* BioParticles and viewed by confocal microscopy. Images are representative of two MDMs and two BMDMs. Scale bars, 20 μm. (**D**) Phagocytosis assays were performed on freshly drawn blood from *Csf1r*-EGFP sheep and wild type controls using pHrodo Red *E.coli* BioParticles. CD14^+^ monocytes were gated in the PBMC fraction by flow cytometry. Results are representative of four wild type and six *Csf1r*-EGFP sheep. The control contained BioParticles and was incubated on ice. (**E**) Representative histograms of MHC class II expression in CD14^+^ PBMCs from wild type (*n* = 5) and *Csf1r*-EGFP (*n* = 7) sheep, analyzed by flow cytometry. (**F**) Representative histograms of MHC class II expression in CD14^+^ and CD16^+^ (GFP^hi^) monocytes from *Csf1r*-EGFP sheep (*n* = 5), analyzed by flow cytometry.

MacGreen (*Csf1r*-EGFP) mice express the transgene in the same locations as the endogenous gene (4). To verify whether expression of the transgene in sheep was restricted to cells expressing Csf1r, we used fluorescently labeled CSF1-Fc (6) to detect Csf1r in whole blood via flow cytometry. Both EGFP^hi^ and EGFP^lo^ PBMCs bound the labeled CSF1-Fc, whereas the EGFP^−^ PBMCs did not (Fig. 6A). Surprisingly, the granulocyte population showed low levels of CSF1-Fc binding. Murine granulocytes from the MacGreen mouse express *Csf1r* mRNA and are EGFP^+^, yet they do not express Csf1r on the cell surface (39). There is evidence that human granulocytes express functional CSF1R, albeit at lower levels than CD14^+^ monocytes (40). We examined EGFP expression in sheep granulocytes. Consistent with the level of CSF1-Fc binding, and identical to the levels of CSF1R in humans, this population expressed low levels of EGFP (Fig. 6A). Expression of endogenous *Csf1r* was not affected by the transgene; the level of mRNA detected by quantitative PCR in BMDMs from wild type and transgenic sheep was not significantly different (Fig. 6B).

BM and PBMCs from *Csf1r*-EGFP sheep differentiated into macrophages in the presence of rhCSF1 (Figs. 4, 5). Phagocytosis assays were performed to further investigate whether the transgene had any effect on monocyte and macrophage function in *Csf1r*-EGFP sheep. Both monocyte-derived macrophages (MDMs) and BMDMs phagocytosed Zymosan A *S. cerevisiae* BioParticles within 2 h (Fig. 6C), highlighting that they were indeed functional macrophages. To compare the function of monocytes from wild type and *Csf1r*-EGFP sheep, we performed phagocytosis assays on whole blood. There was no difference in the capability of CD14^+^ monocytes to phagocytose *E. coli* (Fig. 6D), suggesting that the transgene also had no effect on the function of monocytes.

The development of mature APCs relies on the expression of surface MHC class II molecules. We assessed the expression in wild type and transgenic monocytes via flow cytometry and found that both groups had CD14^+^ monocytes that expressed MHC class II (Fig. 6E). In humans, the highest expression levels of MHC class II are found on CD16^+^ monocytes (30). As in humans, all monocytes express high levels of MHC class II and it is unaffected in the *Csf1r-*EGFP sheep. Again, in common with humans, the CD16^+^ monocytes expressed higher levels than the CD14^++^ subset.

## Discussion

CSF1 controls the proliferation, differentiation, and survival of monocytes and macrophages and their BM progenitors (41), and the receptor, CSF1R, provides a marker for cells of the mononuclear phagocyte lineage. MacGreen (*Csf1r*-EGFP) mice express the transgene in the same locations as the endogenous gene, and expression is dependent on FIRE (4). FIRE acts as an antisense promoter in macrophages (42) and is remarkably conserved from humans to reptiles (43). It is even more highly conserved than the proximal promoter. We have recently described a lentivirus containing control elements of murine *Csf1r* capable of driving transgene expression in macrophages from multiple species, including sheep, in vitro (12). This lentivirus was used in the generation of *Csf1r*-EGFP transgenic sheep. Alignment of the Ensembl predicted sheep *Csf1r* gene with the murine promoter, and FIRE reveals 50 and 80% identity, respectively (data not shown). All of the myeloid-expressed transcription factor binding sites contained within FIRE (44) are conserved between mouse and sheep. Our data show that the apparent conservation reflects function in that the mouse elements were sufficient to drive EGFP expression in monocytes from all four *Csf1r*-EGFP founder sheep.

The *Csf1r* promoter region in mice contains two separate promoters to drive expression in macrophages and placental trophoblast cells (45). In humans, the trophoblast promoter is located 26 kb upstream of *CSF1R* (46). When the human trophoblast promoter sequence is aligned against the sheep genome (Oar_v3.1/oviAri3) using BLAST-like alignment tool, the highest scoring match is located at the 3′ end of *Pdgrfb*. This suggests the ovine trophoblast promoter, as in humans, is located at least 20 kb upstream of *Csf1r*. Deletion of the trophoblast promoter in the MacBlue mice abolishes transgene expression in the majority of tissue macrophages (10). Hence the lack of EGFP expression in AMs from the transgenic sheep may be caused by the lack of the trophoblast promoter in the construct used to generate these animals. We have previously shown that sheep macrophages are EGFP^+^ after incubation with *Csf1r*-EGFP lentivirus, which used polybrene to increase transduction efficiency (12). The low levels of EGFP expression in *Csf1r-*EGFP sheep could likely be increased by the use of a higher titer lentivirus (and hence more lentiviral insertion events) or by use of the ovine instead of murine *Csf1r* in the lentiviral construct. However, it is also possible that the reduced expression level in sheep versus mouse is associated with either species- or lentiviral-specific methylation of the transgene. A study of EGFP^+^ sheep generated by lentiviral injection of zygotes using a ubiquitous promoter indicated increased methylation patterns correlated with lower EGFP intensity (47).

Monocytes are a population of leukocytes that can be functionally characterized by their ability to phagocytose, produce cytokines, and present Ag. They make up 5–10% of the PBMCs in humans and show both antigenic and morphological (size, granularity, and nuclear morphology) heterogeneity. Their initial identification was based on expression of CD14; however, variation in surface expression of Ags has led to the description of various subsets (reviewed in Ref. 48). Human and bovine monocytes can be divided into subpopulations based on surface expression of CD14/CD16, whereas porcine and murine monocytes can be identified by CD14/CD163 and Ly6C/CX3CR1, respectively (reviewed in Ref. 19). The majority of knowledge about monocyte development in the BM stems from work performed in mice (reviewed in Ref. 49). Monocytes develop from hemopoietic stem cells in the BM via a series of progenitors such as the common myeloid progenitor, monocyte-macrophage dendritic cell progenitor, and the common monocyte progenitor, which all express *Csf1r* (50–53). These progenitors can be identified as Lin^−^ and by differential expression of CD117 (c-kit), CX_3_CR1, Ly6C, and CD31. In humans, the common myeloid progenitor can be distinguished from other progenitors based on expression of CD38, CD45RA, FLT3, CD7, and CD10 (54). In-depth studies of monocyte development in larger animals have been hampered by the lack of species-specific Abs. In pigs, the Swine Workshop Cluster molecules were originally used to identify committed monocyte precursors in the BM. Swine Workshop Cluster 3 was identified as the earliest marker of myeloid cell development (55) and was later identified as CD172a (56). In cows and sheep, CD34^+^ progenitor cells have been identified (57, 58), yet specific analysis of monocyte progenitors has not been performed.

As noted earlier, the expression of the EGFP transgene in the sheep is downregulated by CSF1 in vitro and in tissue macrophages in vivo. Tissue macrophages depend upon CSF1 for their differentiation, and they are rapidly depleted after treatment of mice with anti-CSF1R Ab (5). The downregulation of the reporter gene by CSF1 probably reflects direct actions on FIRE. FIRE enhancer activity is acutely regulated by transcription factor Runx1, which is expressed at high levels in progenitors and acutely downregulated by CSF1 (59). There is an emerging view that most tissue macrophages are replaced by self-renewal, rather than replacement from the blood monocyte pool (60, 61). Like the MacBlue transgene in mice, which is effectively monocyte specific (62, 63), the *Csf1r*-EGFP lentiviral transgene could provide a useful marker to monitor monocyte extravasation and trafficking in tissues, and might be applied to other species.

Mice and humans differ immunologically, and the mouse has limitations as a model (64–66). Macrophages from the pig more closely resemble those of humans in terms of their response to bacterial LPS (21) or sheep in terms of pattern recognition receptor expression (67). Hence larger animal models are likely to represent a better model of human macrophage development. Phenotypic analysis of macrophage development in sheep has not been described. Instead, studies have focused on the response of monocytes and macrophages to infection. We have shown that monocytes from *Csf1r*-EGFP sheep are functional and comparable with their wild type counterparts, and that the transgene had no effect on expression of the endogenous gene. Hence, as expression of *Csf1r* is one of the earliest markers of macrophage lineage commitment (51), BM from *Csf1r-*EGFP sheep could be a valuable resource to study the earliest events in myelopoiesis in an underused species.
